# Transcriptional study reveals a potential leptin-dependent gene regulatory network in zebrafish brain

**DOI:** 10.1007/s10695-021-00967-0

**Published:** 2021-07-08

**Authors:** Ehsan Pashay Ahi, Emmanouil Tsakoumis, Mathilde Brunel, Monika Schmitz

**Affiliations:** 1 Department of Organismal Biology, Comparative Physiology, Evolutionary Biology Centre, Uppsala University, Norbyvägen 18A, SE-752 36 Uppsala, Sweden; 2 Organismal and Evolutionary Biology Research Programme, University of Helsinki, Viikinkaari 9, 00014 Helsinki, Finland; 3 Department of Molecular Sciences, Swedish University of Agricultural Sciences, Allmas Allé 5, SE-750 07 Uppsala, Sweden

**Keywords:** Leptin receptor, Zebrafish, Gene expression, Gene regulatory network, Feeding, Brain

## Abstract

The signal mediated by leptin hormone and its receptor is a major regulator of body weight, food intake and metabolism. In mammals and many teleost fish species, leptin has an anorexigenic role and inhibits food intake by influencing the appetite centres in the hypothalamus. However, the regulatory connections between leptin and downstream genes mediating its appetite-regulating effects are still not fully explored in teleost fish. In this study, we used a loss of function leptin receptor zebrafish mutant and real-time quantitative PCR to assess brain expression patterns of several previously identified anorexigenic genes downstream of leptin signal under different feeding conditions (normal feeding, 7-day fasting, 2 and 6-h refeeding). These downstream factors include members of *cart* genes, *crhb* and *gnrh2*, as well as selected genes co-expressed with them based on a zebrafish co-expression database. Here, we found a potential gene expression network (GRN) comprising the abovementioned genes by a stepwise approach of identifying co-expression modules and predicting their upstream regulators. Among the transcription factors (TFs) predicted as potential upstream regulators of this GRN, we found expression pattern of *sp3a* to be correlated with transcriptional changes of the downstream gene network. Interestingly, the expression and transcriptional activity of *Sp3* orthologous gene in mammals have already been implicated to be under the influence of leptin signal. These findings suggest a potentially conserved regulatory connection between leptin and *sp3a*, which is predicted to act as a transcriptional driver of a downstream gene network in the zebrafish brain.

**Supplementary Information:**

The online version contains supplementary material available at 10.1007/s10695-021-00967-0.

## Introduction

Feeding behaviour is controlled by processes involving appetitive behaviours (hunger-driven activities) and food ingestion (Keen-Rhinehart et al. 2013). The central regulation of feeding behaviour in brain (hypothalamus region) is influenced by endocrine signals, which are triggered after exposure to different metabolic and nutritional conditions. The neurons mediating the appetite-regulating effects, so called accurate nucleus neurons in mammals (Opazo et al. 2019), are located in the periventricular and lateral parts of the hypothalamus in fish (Jeong et al. 2018). These neurons can be classified into two main types: orexigenic, stimulating food intake and/or locomotor activity, and anorexigenic, reducing food intake and/or locomotor activity (Sohn 2015). The appetite-regulation genes are the major player in these processes, and to this date, a range of neuropeptides and their cognate receptors encoded by these genes are identified in fish (Volkoff 2016). These genes can also be categorised based on their orexigenic and anorexigenic functions (Arora and Anubhuti 2006; Parker and Bloom 2012). Despite recent advances in understanding the functions of these genes in controlling appetite, little is known about their transcriptional regulatory connections under different feeding conditions in fish.

In mammals, leptin has been shown to be an upstream transcriptional stimulator of several anorexigenic genes in the brain, such as *Cart*, *Crh*, *Mc4r*, *POMC* (Schwartz et al. 1996; Thornton et al. 1997; Ghamari-Langroudi et al. 2011; Lee et al. 2013), suggesting that leptin mediates its effects on feeding behaviour through induction of these genes in the brain. However, in fish, similar positive regulatory connections have only recently been reported between leptin signal and transcription of these anorexigenic genes in zebrafish (Ahi et al. 2019a). In general, little is known about leptin-dependent regulatory mechanisms in fish and previous attempts to reveal the complexity of leptin mediated transcriptional regulation of biological processes have mainly addressed its other physiological roles (e.g. its role in mechanisms controlling fish reproduction (Paolucci et al. 2020; Wang et al. 2020)).

The leptin-dependent phenotypic effects on fish growth remain still controversial. In medaka, a loss of function mutation in leptin receptor (*lepr*) has shown to increase food intake and growth rate at the post-juvenile stage (Chisada et al. 2014). In zebrafish, however, while a loss of function mutation in *lepa* (one of the two genes encoding leptin) causes similar effects such as increased weight and length under normal feeding condition (Audira et al. 2018), different mutations in the *lepr* gene result in controversial phenotypes (Michel et al. 2016; Fei et al. 2017). For example, with a mutation causing a premature termination codon in *lepr*, the adult zebrafish did not exhibit any growth changes under normal feeding and overfeeding conditions (Michel et al. 2016). Whereas when introducing a 17 bp deletion in *lepr* gene, the adult zebrafish showed increased weight under overfeeding conditions (Fei et al. 2017).

In our previous study, we found no phenotypic difference under normal feeding condition, using a different loss of function zebrafish *lepr* mutant (Ahi et al. 2019a). However, based on our recent observations, the same zebrafish *lepr* mutant showed significant increase in length and weight under overfeeding (unpublished data). At transcriptional level, we found decreased expression of several anorexigenic genes in the brain of zebrafish carrying a non-functional mutant of leptin receptor gene (Ahi et al. 2019a). Among these anorexigenic genes, we found consistent reduced expression of all the cocaine- and amphetamine-regulated transcripts, *cart* genes, as well as their potential downstream target gene, *crhb* (corticotropin-releasing hormone), in the leptin receptor mutant (*lepr*^−/−^) at normal feeding condition (Ahi et al. 2019a). The anorexigenic role of *cart* genes is suggested in zebrafish (Nishio et al. 2012; Guillot et al. 2016) and other teleost fishes (Volkoff 2016). However, not all *cart* genes have similar expression distribution in the zebrafish brain, and they do not follow similar expression patterns in response to changes in feeding conditions suggesting a complex expression regulation (Akash et al. 2014). In goldfish, another member of Cypriniformes, only *cart1* inhibition of feeding is regulated by leptin in the brain (Volkoff and Peter 2001). A similar regulatory connection between *cart* and leptin is also observed in an evolutionary distant catfish species (Subhedar et al. 2011). Therefore, our finding of similar expression changes of all cart genes in response to the absence of leptin signal suggests the potential existence of a shared leptin-dependent upstream regulator in the zebrafish brain. On the other hand, the similar expression differences of *crhb*, which also has anorexigenic effects in fish (De Pedro et al. 1993; Bernier 2006), confirms potential regulatory connections between *cart* genes and *crhb* in zebrafish brain, as observed both in mammalian and avian brains (Sarkar et al. 2004; Smith et al. 2004; Mo et al. 2015). Furthermore, the decreased expression of *crhb* was accompanied with reduced expression of *gnrh2*, a member of gonadotropin-releasing hormones with anorexigenic function in zebrafish and goldfish brain (Hoskins et al. 2008; Nishiguchi et al. 2012). Interestingly, *gnrh2* is shown to be a direct downstream target of *crh* in goldfish (Kang et al. 2011) and similar expression patterns of *crh* and *gnrh2* in response to feeding were recently reported in another Cypriniformes species (*Schizothorax davidi*) (Yuan et al. 2021). These suggest the presence of a potentially conserved *cart-crhb-gnrh2* regulatory axis at downstream of the leptin signal in zebrafish brain (Ahi et al. 2019a). Nevertheless, the detailed regulatory mechanisms linking the expression of *cart, crhb* and *gnrh2* genes as well as their connection to leptin signal remain unclear in vertebrates.

With the advent of ever-growing databases for gene co-expression networks in a variety of organisms, including zebrafish (e.g. see (Obayashi et al. 2019)), as well as prediction tools for transcription factor binding sites (e.g. TRANSFAC (Matys et al. 2003)), it becomes possible to identify gene regulatory networks through examining the expression of members of a predicted network. In this study, we aimed to investigate the existence of potential GRN(s) containing *cart*, *crh* and *gnrh2* genes, which can be controlled by leptin signal in zebrafish brain under different feeding conditions. For this, we followed a simple stepwise gene detection approach using qPCR in order to deduce GRNs involved in various biological processes in fish (Ahi et al. 2015; Ahi and Sefc 2018). Based on this approach, we used a zebrafish co-expression database to select the top ranked co-expressed genes with our genes of interest (*cart*, *crh* and *gnrh2*) to assess their expression pattern by qPCR. We first identified gene modules co-expressed with *cart*, *crh* and *gnrh2* genes, and next we predicted their potential upstream transcriptional regulators. Based on our gene expression results, we predicted GRNs, at downstream of leptin signals in zebrafish brain, which might be affected by changes in feeding and contribute to leptin-dependent metabolic and behavioural effects. Our findings provide first evidence for environmentally influenced GRNs, which might be directly controlled by leptin signalling in the brain of a vertebrate species.

## Methods

### Zebrafish husbandry

Zebrafish belonging to the strain LepR Sa12953 were obtained from the European Zebrafish Resource Centre. The mutation of the *lepr* gene was created by the Sanger Institute for the Zebrafish Mutation Project, replacing a thymine with an adenine on chromosome 6, resulting in a premature stop codon and thus to a shortened polypeptide. Wild-type and *lepr* mutant zebrafish of similar age were kept in 3-l recirculating tanks, under an artificial photoperiod of 14/10 light/dark conditions at 28.4 °C at the Genome Engineering Zebrafish National Facility at Uppsala University (or SciLife lab).

### Experimental set-up and sampling of tissues

Detailed description of the experimental set-up can be found in Ahi et al. (2019a). Briefly, siblings wild-type and *lepr* mutant zebrafish fish were randomly selected at the beginning of the experiment from different stock tanks of fish and put in a 3-l tank together for each genotype and for each feeding category: fish fed normally (control group); fish fasted for a week; fish fasted for a week and sampled 2 h after refeeding; and fish fasted for a week and sampled 6 h after refeeding (Supplementary data 1). Each 3-l tank contained 5 fish with mix female/male ratios (1–2 females and 3–4 males) depending on the numbers of males and females available for each genotype at that moment. Tanks were placed next to each other and were connected to the same water system, indicating that the water parameters were exactly the same for all tanks as well as the sanitary measures taken to avoid diseases (filters and UV light). The water flow was set at the same speed manually for each tank and light conditions were comparable for all tanks as they were located at the same place. Water parameters were regularly monitored by the facility staff. Specifically, water temperature (°C), pH and conductivity (μS/cm) were measured daily, while general hardness (°dGH) and carbonate hardness (°dKH), as well as the levels of ammonia (NH4, mg/l), nitrites (NO_2_, mg/l) and nitrates (NO_3_, mg/l) were measured bi-weekly.

During the experiment, fish were fed once a day with dry pellets (morning) and twice with *Artemia* (middays and evenings). No significant differences were observed in the standard body length, the net weight and the hepato-somatic index (HSI) between the two genotypes (Supplementary data 1). During the sampling, fish were first anaesthetized by immersion in a 0.4 mg/ml Tricaine (MS-222) solution and euthanatized by immersion in ice bath. Zebrafish were decapitated, their brains were carefully dissected, transferred into 200 μl RNAlater RNA extraction stabilisation solution (Ambion Inc., Austin, TX) and stored at 4 °C for 24 h and then at − 20 °C until the RNA isolation step.

### RNA isolation and cDNA synthesis

The total RNA of the sampled brain tissues was extracted using Trizol (Ambion), according to the manufacturer’s protocol. Briefly, the dissected brains were removed from RNAlater and were homogenised in 200 μl Trizol, with a fine syringe needle (25G Terumo needle and BD Plastipak 1-ml syringe). Once they were thoroughly homogenised, 40 μl of chloroform (Sigma-Aldrich) were added to each sample, followed by a 5-min incubation in room temperature and a centrifugation at 12,000 g/min for 20 min at 4 °C. The aqueous upper phase was then carefully transferred into new RNAse-free tubes, in which 1 μl of glycoblue (Ambion) and 100 μl of ice cold (− 20 °C) isopropyl alcohol (Sigma-Aldrich) were added directly. Samples were mixed rigorously, incubated for 5 min at room temperature and then centrifuged at 13,000 g/min for 20 min at 4 °C. The supernatants were discarded and the RNA pellets were washed three times with 200 μl of ice cold (− 20 °C) 75% ethanol solution (VWR) by centrifugation at 9000 g/min for 5 min at 4 °C. The RNA pellets were dried under the fume hood for 10 min at room temperature and were solubilised, by adding 10 μl of nuclease-free water (Ambion). All the RNA samples were afterwards DNAse treated, to remove any genomic DNA contamination, using the Turbo DNA-free kit (Ambion), according to the manufacturer’s instructions. The quantity and quality of the final RNA samples were measured spectrophotometrically, using NanoDrop (Thermo-Fisher Scientific). cDNA synthesis was carried out by reverse transcription (RT) with 1000 ng of RNA input from each sample. Specifically, 0.5 μl of random primers (50 ng/μl) and 0.5 μl dNTP (10 nM) were added in each RNA sample, following an incubation at 65 °C for 5 min and then the samples were cooled down on ice for 1 min. A total mix of 3.5 μl, containing 2 μl of 5X First-Strand Buffer, 0.5 μl of DTT (0.1 M), 0.5 μl of RNase OUT (40 U/μl) and 0.5 μl of Superscript III RT (200 U/μl), was prepared and added to each sample. The thermal profile of the RT was 25 °C for 5 min, 50 °C for 50 min and 70 °C for 15 min. The final volume of 10 μl of cDNA from each sample was stored at − 20 °C until further analysis.

### Gene selection, primer design and qPCR

We followed an already described approach of gene regulatory network deduction using zebrafish co-expression data available at COXPRESdb (http://coxpresdb.jp/) version 7.0 (Obayashi et al. 2019). To do this, we first selected 5 top ranked genes with highest co-expression values with all of the *cart* genes (*cart1-4*) in zebrafish, and the same was performed for selection of top 5 genes co-expressed with *crhb* and *gnrh2*. The selection criteria was limited to only the genes with supportability score of 1 as minimum (described in COXPRESdb (Obayashi et al. 2019)) (Supplementary data 1). Among the selected candidate genes, those showing expression differences similar to *cart* genes (first co-expression module) or to *crhb* (second co-expression module) were chosen for the next step of upstream regulator prediction. We performed motif enrichment on 4-kb upstream sequences (promoter and 5′-UTR) of these genes (for the identified genes in each module separately) using MEME algorithm (Bailey et al. 2009). The overrepresented motifs in the promoters of the genes were compared to position weight matrices (PWMs) obtained from the TRANSFAC database (Matys et al. 2003) using STAMP (Mahony and Benos 2007) to identify potential transcription factor (TF) binding sites.

Specific primers for each target and reference gene were designed, based on the genes sequences obtained from Blastn through a zebrafish database engine (zfin.org) (Howe et al. 2013). The sequences were imported to the CLC Genomic Workbench (CLC Bio, Denmark), and the exon/exon boundaries were tracked using the annotated *Danio rerio* genome in the Ensembl database (Flicek et al. 2012). Primers with short amplicon sizes (< 200 bp) were designed using the Primer Express 3.0 software (Applied Biosystems, CA, USA) and their dimerization and secondary structure formation were lastly evaluated using OligoAnalyzer 3.1 (Integrated DNA Technology) (Supplementary data 1).

Relative gene expression levels were measured by quantitative polymerase chain reaction (qPCR) on a MxPro-3000 PCR machine (Stratagene, La Jolla, CA), using the MxPro software (Stratagene) for data mining. For qPCR assays, 1 μl of diluted cDNA of each sample was mixed with 7.5 μl of qPCR PowerUp SYBR Green Master mix (Thermo-Fisher Scientific), 0.3 μl of forward and reverse primers (10 µM) and 6.2 μl of RNA-free water in a total volume of 15 μl. Each biological replicate was tested in two technical replicates for each gene, followed by a sample maximisation method (Bustin et al. 2009), aiming to have an optimal experimental set-up in each run. The thermal profile of the qPCR assays was 50 °C for 2 min (1 cycle), 95 °C for 2 min (1 cycle), 95 °C for 15 s and 62 °C for 1 min (40 cycles). A dissociation step (60–95 °C) was also performed after the amplification step, to verify the specificity of the product. For the calculation of the primer efficiencies, standard curves were generated using serial dilutions of pooled cDNA of random samples obtained from the RT step and were tested in three technical replicates. Standard curves were calculated using the following formula: E = 10[− 1/slope]. R^2^ values were higher than 0.990 and efficiencies were ranging between 94 and 108% for all assays (Supplementary data 1).

### Gene expression analysis

In this study, we used the Cq values of a validated reference gene, glucose-6-phosphate dehydrogenase (*g6pd*), showing stable brain expression across both genotypes and different feeding conditions to normalise Cq values of target genes for each sample (ΔCq_target_ = Cq_target_ − Cq_reference_) (Ahi et al. 2019a). We selected a biological replicate with lowest expression (highest Cq value) for each gene and then used the following formula; ΔCq_target_ − ΔCq_calibrator_, in order to calculate ΔΔCq values. The relative expression quantities (RQ values) were calculated as 2^−ΔΔCq^ and their logarithmic values (fold changes) were applied for statistical analysis (Pfaffl 2001). Student t-tests were carried out for the direct comparison of the gene expression levels for each target gene between the two genotypes in each feeding condition. Analysis of variance (ANOVA) tests, followed by Tukey’s honest significant difference (HSD) post hoc tests, were performed between the different feeding conditions within each genotype, for the analysis of the dynamic expression of the target genes. Benjamini–Hochberg procedure was used to correct the false positive rate in the multiple comparisons (Thissen et al. 2002). In order to search for any similarities in the expression patterns across the feeding conditions in each genotype, we performed pairwise Pearson correlation coefficients. Finally, in order to identify overall similarities between the different feeding conditions and genotypes, we implemented a dendrogram hierarchical clustering of the expression values of the target genes. All statistical analyses were carried out using the R software (http://www.r-project.org) (Team RDC 2013).

## Results

### Expression analyses of *cart* 1–4, *crhb* and *gnrh2* co-expressed genes

**Fig. 1:**
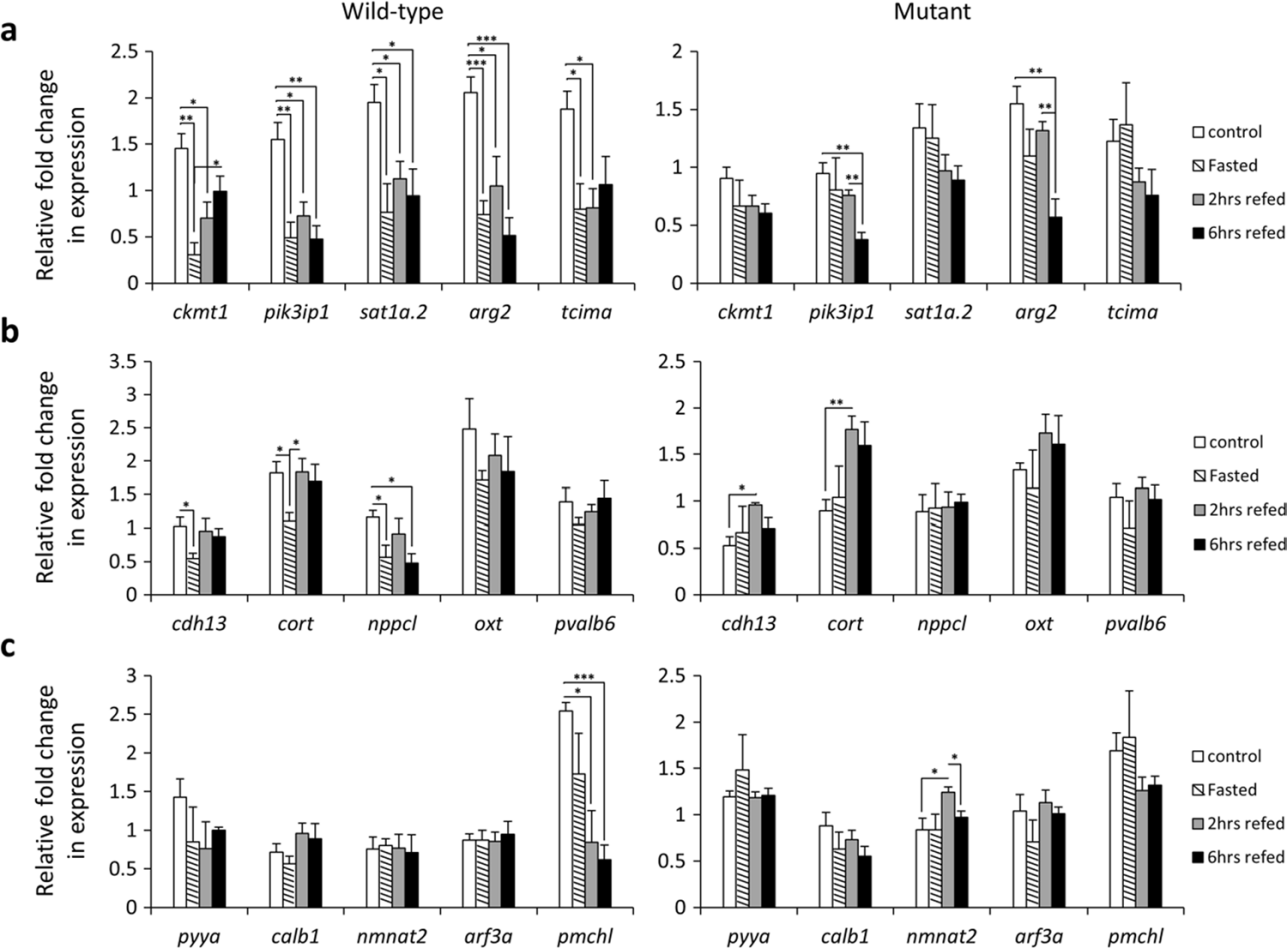
Expression dynamics of selected co-expressed genes within each genotype during the fasting-refeeding experiment. Expression changes of (**a**) *cart*-co-expressed genes, (**b**) *crhb*-co-expressed genes and (**c**) *gnrh2*-co-expressed genes within each genotype. Means and standard errors of fold changes in expression of five biological replicates are shown for each experimental group. Significant differences are indicated by asterisks (*P < 0.05; **P < 0.01; ***P < 0.001)

**Fig. 2:**
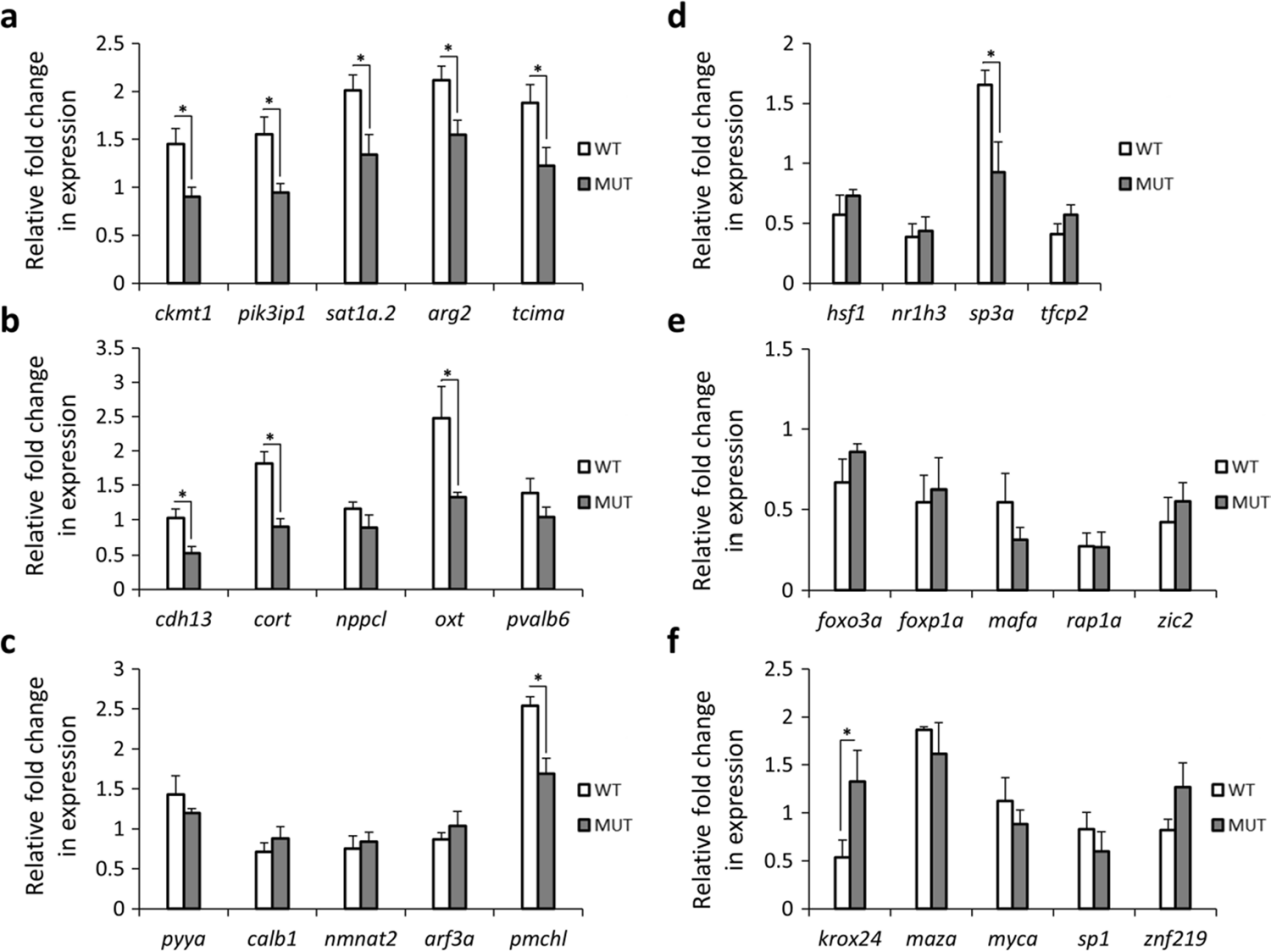
Gene expression differences between the two genotypes, wild-type (WT) and *lepr* mutant, at normal feeding condition. (**a**–**c**) Differential expression of genes co-expressed with *cart1-4*, *crhb* and *gnrh2*. (**d**–**f**) Predicted TFs at upstream of *cart1-4*, *crhb* and *gnrh2* co-expressed modules. Means and standard errors of fold changes in expression of five biological replicates are shown for each experimental group. Significant differences are indicated by asterisks (*P < 0.05)

In order to identify a gene co-expression module which includes *cart* genes, we followed a knowledge-based and stepwise approach previously established to identify GRNs linked to phenotypic differences in teleost fish (Ahi et al. 2015; Ahi and Sefc 2018). The first step was to use a zebrafish co-expression database, COXPRESdb (Obayashi et al. 2019), in order to select candidate genes with potential co-regulatory connections to *cart* genes. To do this, we selected top 5 genes with highest probability of expression correlation with all the four *cart* genes in zebrafish co-expression database (Supplementary data 1). Expression profiling of these genes within each genotype revealed that in wild-type zebrafish changes in normal feeding conditions (after fasting and refeeding) reduce their expression in the brain and interestingly this pattern was almost lost in the *lepr* mutant (Fig. 1a). The direct comparison of the two genotypes within each treatment group demonstrated that in the *lepr* mutant the expression of all 5 genes (*ckmt1*, *pik3ip1*, *sat1a.2*, *agr2* and *tcima*) is reduced under normal feeding conditions but not after changes in the feeding condition (Fig. 2a and Supplementary data 1). This could imply that fasting and refeeding might trigger other molecular signal(s) which override the leptin-dependent differential regulation of these genes. Although, a higher number of samples are required to assure that the absence of the signal is because of the increase of variation in response to fasting or of the existence of an overriding signal triggered by fasting. It should be noted that the expression dynamics of the 5 *cart*-co-expressed genes were similar to the expression patterns of *cart* genes under similar conditions, i.e. reduced expression in the *lepr* mutant in the normal feeding group (Ahi et al. 2019a). This suggests potential co-regulatory connections between *cart* genes and the 5 selected co-expressed genes as predicted in COXPRESdb (Obayashi et al. 2019).

We also conducted the same approach to identify *crhb* co-expressed genes by selecting top 5 genes with highest probability of expression correlation with *crhb* in zebrafish database. We found 3 genes, *cdh13*, *cort* and *nppcl*, with reduced expression (similar to *crhb*) in fasting group compared to the control group in wild-type, and such expression pattern was not present in *lepr* mutant (Fig. 1b). The direct comparison of the two genotypes within each treatment group showed that again only 3 genes, *cdh13*, *cort* and *oxt* (but not *nppcl*), had higher expression in wild-type than the mutant zebrafish under normal feeding condition (control group) (Fig. 2b). Altogether, these findings indicate that the 4 genes *cdh13*, *cort*, *nppcl* and *oxt* show similar expression pattern to *crhb* as identified in our previous study (Ahi et al. 2019a), suggesting that partial co-regulatory connections between *crhb* and the selected co-expressed genes have been retained in zebrafish brain.

Finally, after applying the same approach for *gnrh2* co-expressed genes (the top 5 genes based on the zebrafish database), only one of the genes, *pmchl*, showed fairly similar expression pattern to *gnrh2* in the wild-type group (Fig. 1c). The same gene also showed higher expression in wild-type compared to the mutant under normal feeding condition (Fig. 2c). This suggests that only the co-regulatory connection between *gnrh2* and *pmchl* out of the top 5 selected co-expressed genes has been retained in the zebrafish brain.

### Expression analyses of predicted upstream regulators of the co-expression modules

In a next step, we searched for potential upstream regulators of each of the identified gene co-expression modules through prediction of TF binding sites in the upstream regulatory sequences of the co-expressed genes. For the *cart* co-expression module, we used promoter and 5′-UTR sequences of all *cart* genes and the 5 validated co-expressed genes (*ckmt1*, *pik3ip1*, *sat1a2*, *arg2* and *tcima*) for motif enrichment analysis step. We found 6 motifs present in the regulatory sequences of these genes (Table 1) and after parsing the motifs against the vertebrate TF binding sites, we listed top matched TF(s) for each motif (Table 1). Similarly, we retrieved the regulatory sequences of *crhb* and 4 of its co-expressed genes *cdh13*, *cort*, *nppcl* and *oxt*. The enrichment analysis yielded 5 motifs which could be matched to TF binding sites as listed in Table 1. Finally, the analysis on the regulatory sequences of *gnrh2* and *pmchl* identified 4 motifs, which could be matched to 6 TF binding sites (Table 1). Only one of the TF binding sites, FOXP1, was shared between the predicted upstream TFs of *cart* and *crhb* co-expression modules. On the other hand between *gnrh2* and *crhb* co-expression modules only RAP1 binding site was found to be shared.

**Table 1:** Predicted TF binding sites for potential upstream regulators of the three gene coexpression modules. PWD ID indicates positional weight matrix ID of a predicted binding site and E-values refer to matching similarity between the predicted motif sequences and the PWD IDs. The count implies on number of genes in each network containing the predicted motif sequence on their regulatory region

	TF binding site	PWM ID	Count	Predicted motif sequence	E-value
*cart* network	LXR (Nr1h3)	M00766	9/9	CRCCCGBMDGAAACCCACVCAMACGCASSGAG	5.00E − 09
	FOXP1	M00987	9/9	AWAWAWATAWATAWATAAATAAATAAAW	1.39E − 08
	AIRE	M00999	9/9	AWAWAWATAWATAWATAAATAAATAAAW	1.70E − 08
	AIRE	M01000	8/9	TYATTTTATTTATTKTAHATTWTTTTTGT	3.03E − 08
	SP3	M00665	9/9	CMMTTKGASAGGKCAKWGG	1.67E − 07
	TFCP2 (LSF)	M00947	7/9	CTGRCCYAGMCKSGGCTSRARCCAGYGAC	7.26E − 07
	HSF1	M00163	9/9	TTYHTTCATTTTCTTTTSBKT	9.09E − 07
*crh* network	ZIC2	M00449	4/5	GGGGYGGTACC	8.65E − 07
	FOXO3	M00477	5/5	TBCTTTGKCTWCATA	1.73E − 06
	MAF	M00648	5/5	CCCMAACBYCMCTYKBKMCTG	2.00E − 06
	RAP1	M00213	5/5	GTGTGTGBGT	3.27E − 06
	FOXP1	M00987	5/5	ACACACACRCACACACAWVKG	8.60E − 06
*gnrh2* network	MAZ	M00649	2/2	GGRAGGGG	1.90E − 09
	SP1	M00931	2/2	GGRAGGGG	4.32E − 08
	ZNF219	M01122	2/2	CCAYCMCSSYCCMCC	6.66E − 08
	KROX	M00982	2/2	CCAYCMCSSYCCMCC	9.33E − 08
	c-MYC	M00123	2/2	CAAAGACATGYGGTACAGGTGAAWTGGGTRKGC	1.53E − 07
	RAP1	M00213	2/2	TGTGTRTGKATGT	1.81E − 07

**Fig. 3:**
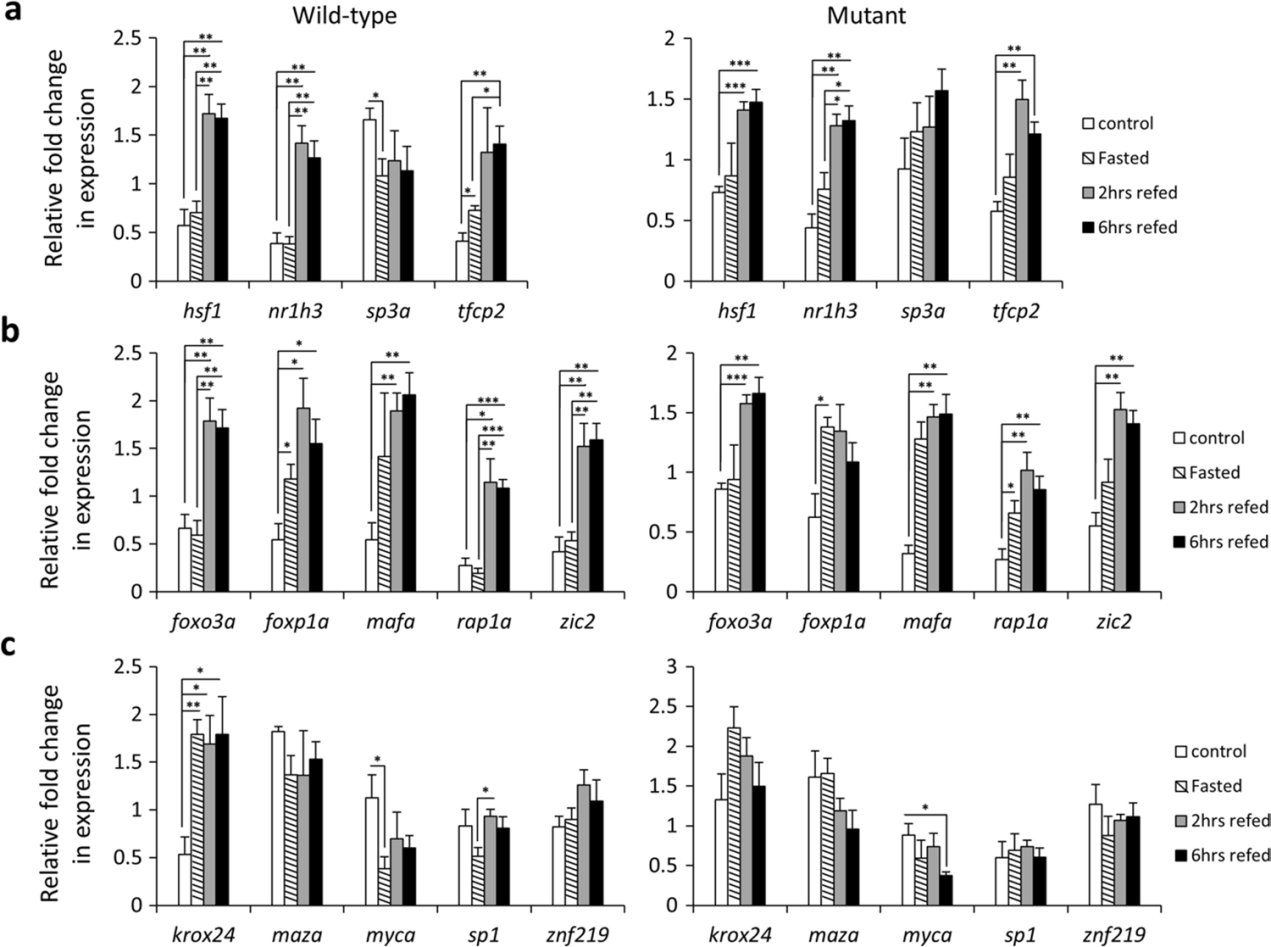
Expression dynamics of predicted upstream regulators of the co-expressed genes within each genotype during the fasting-refeeding experiment. Expression changes of predicted TFs at upstream of (**a**) *cart*-co-expressed genes, (**b**) *crhb*-co-expressed genes and (**c**) *gnrh2*-coexpressed genes within each genotype. Means and standard errors of fold changes in expression of five biological replicates are shown for each experimental group. Significant differences are indicated by asterisks (*P < 0.05; **P < 0.01; ***P < 0.001)

The expression analysis of potential upstream TFs of *cart* co-expression module revealed that 3 of the TFs, *hsf1*, *nr1h3* and *tfcp2*, had similar expression pattern in both genotypes under different feeding conditions with increased expression during refeeding (Fig. 3a). However, only one of the TFs, *sp3a*, followed an expression pattern similar to genes in *cart* co-expression module with higher expression in the control group than the fasting group. Furthermore, in the direct comparison of the genotypes, *sp3a* was the only TF that showed higher expression in wild-type than the mutant in the control group (Fig. 2d). These findings suggest that *sp3a* might act as an upstream transcriptional regulator of *cart* co-expression module and its expression is under the influence of leptin signal during normal feeding condition. The expression profiling of predicted TFs upstream of *crhb* co-expression module did not reveal any TF with similar expression patterns compared to members of the module (Fig. 3b). Instead, all the predicted TFs showed increased expression during refeeding in both genotypes. The direct comparison of the two genotypes within the normal feeding group revealed no expression differences (Fig. 2e). Altogether, the inconsistent expression patterns of predicted TFs and the genes in *crhb* co-expression module raise the possibility that the genes in this module are regulated indirectly through interaction with other upstream regulators than the TFs with binding sites on their regulatory sequences. Among the predicted upstream TFs of *gnrh2* co-expression module, 3 of the TFs, *krox24*, *myca* and *sp1*, had differences in expression pattern within each genotype (Fig. 3c). However, the direct comparison of the two genotypes in the normal feeding group revealed expression difference for only *krox24*, higher expression in the mutant than the wild-type (Fig. 2f). This suggests potential inhibitory effects of *krox24* on transcription of *gnrh2* and *pmchl* in zebrafish brain at normal feeding condition.

### Expression correlation analyses revealing a gene regulatory network

**Fig. 4:**
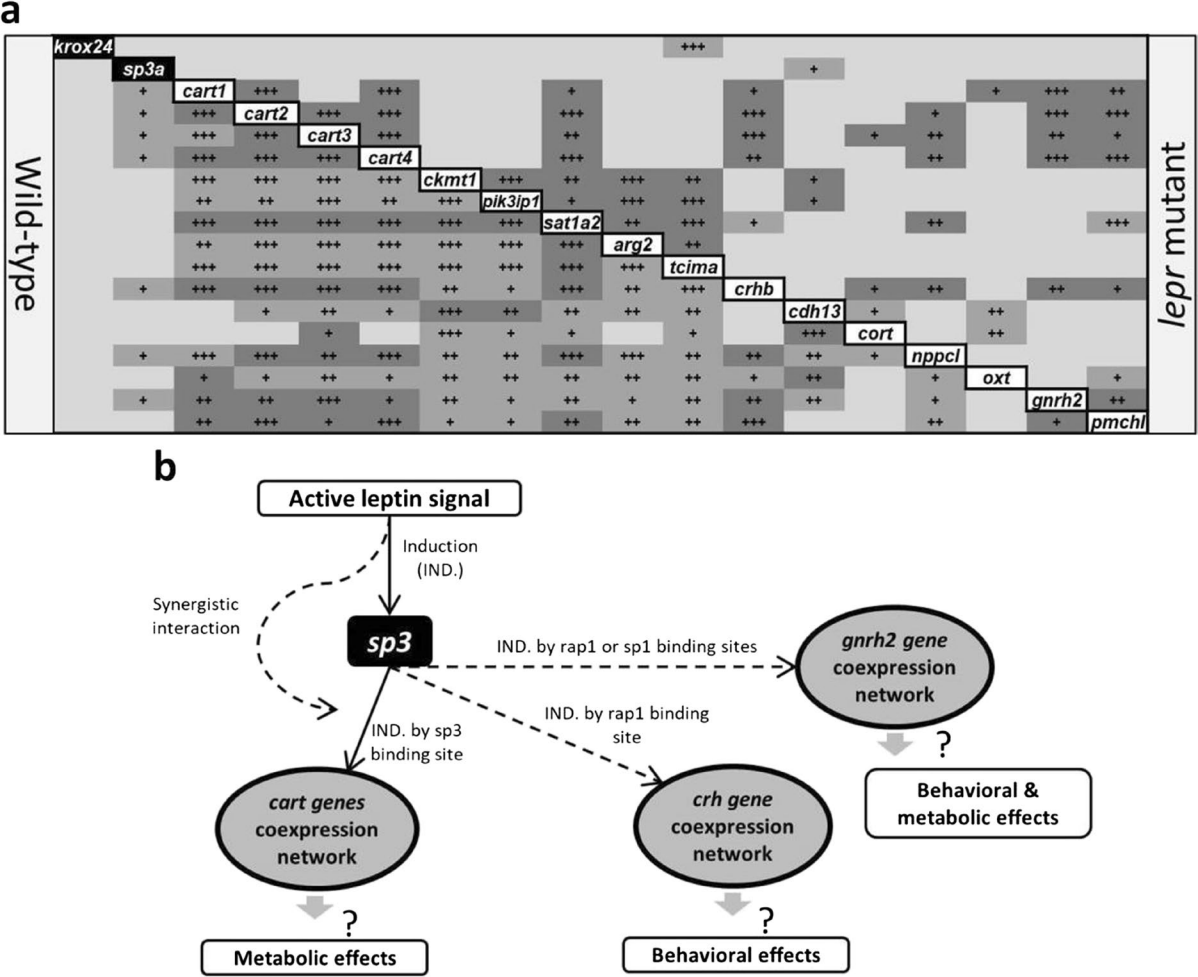
Expression correlations of *cart*, *crhb* and *gnrh2* related gene regulatory networks and their potential regulatory connections. (**a**) Pairwise expression correlations between the members of the *cart* related gene regulatory network in the brain of wild-type and *lepr* mutant zebrafish in the fasting-refeeding experiment. The plus signs indicate positive Pearson correlation coefficients, and 1 to 3 signs represent significant levels of *P* < 0.05, *P* < 0.01 and *P* < 0.001. The pairwise correlations delineated with black borders are similar between the two genotypes. (**b**) A schematic illustration of a potential regulatory interaction between leptin signal and *cart*-/*crh*-/*gnrh2*-coexpression modules mediated by *sp3a* in zebrafish brain

Within each genotype, we performed pairwise expression correlation analysis between *cart*, *crhb* and *gnrh2* co-expressed genes, and two of their predicted upstream regulators, *krox24* and *sp3a*, in order to identify potential regulatory connections between them (Ahi and Sefc 2018). In both genotypes, almost all of the observed correlations appeared to be positive; however, not all of the positive correlations were similar between the genotypes and many of the positive correlations were lost in the mutant with impaired leptin signal (Fig. 4a). For example, in the wild-type, all of the *cart* genes showed positive expression correlations with the selected *cart* co-expressed genes forming a complete co-expression module (Fig. 4a). Whereas, in the *lepr* mutant almost all of the positive expression correlations between *cart* genes and their selected co-expressed genes were lost (except for *sat1a2*) indicating potentially weakened co-regulatory connections between them and loss of the co-expression module (Fig. 4a). Among all the predicted upstream regulators, only *krox24* and *sp3a* had shown differential expression in the direct comparison of the genotypes at normal feeding condition. However, *sp3a* was the only TF that showed multiple positive expression correlations with other genes including *cart* family, *crhb* and *gnrh2* (Fig. 4a). These positive correlations were all lost in the *lepr* mutant indicating that *sp3a* is the potential upstream transcriptional inducer of the co-expression modules and its activity depends on functional leptin signalling. Furthermore, *krox24* did not show any expression correlations with the genes in the downstream network indicating a less crucial regulatory role. Altogether, these findings suggest a potentially active regulatory axis of *lepr*-*sp3a*-*cart*/*crhb*/*gnrh2* genes in zebrafish brain.

## Discussion

One of the major findings in our previous study was the consistent and similar expression differences for all of the *cart* genes (cocaine- and amphetamine-regulated transcripts) between the wild-type and *lepr* mutant. All the four *cart* genes showed reduced brain expression after changes in feeding condition but only in the wild-type zebrafish (Ahi et al. 2019a). We demonstrated that fasting reduces the expression of all the *cart* genes in the brain and their expression reduction maintains during short-term refeeding period (i.e. 2 h and 6 h after refeeding). This is in agreement with the conserved anorexigenic role of *cart* genes shown in zebrafish (Nishio et al. 2012; Guillot et al. 2016). It appeared that these transcriptional changes were dependent on a functional leptin signal, raising the question, what potential regulatory network(s) link these processes. In goldfish brain, the *cart1* mediated inhibition of feeding is regulated by leptin signal (Volkoff and Peter 2001), indicating a conserved regulatory connection between active leptin signal and transcription of *cart* genes in both zebrafish and goldfish. The leptin-dependent transcriptional regulation of *cart* genes seems to be a conserved mechanism in vertebrates, as observed in rodents, where leptin administration increases the brain expression of *Cart1* gene (Lee et al. 2013) and non-functional leptin signal leads to absence of *Cart1* expression in the brain (Kristensen et al. 1998).

In addition, similar expression patterns to *cart* genes were also observed for two other anorexigenic genes, *crhb* (corticotropin-releasing hormone) (De Pedro et al. 1993; Bernier 2006) and *gnrh2* (gonadotropin-releasing hormone 2) (Hoskins et al. 2008; Nishiguchi et al. 2012), suggesting regulatory connections between *cart* genes, *crhb* and *gnrh2* in zebrafish brain. In mammals and birds, it had been demonstrated that *CRH* and *CART1* have transcriptional regulatory connections; however, the details of these regulatory mechanisms still remained unclear (Sarkar et al. 2004; Smith et al. 2004; Mo et al. 2015). *CRH* has been shown to act downstream of activated leptin signal in rat and its brain expression increased after leptin administration (Schwartz et al. 1996). Similarly, the *cart*-dependent regulation of *Gnrh* secretion by activated leptin signal had been demonstrated in rat brain as well (Lebrethon et al. 2000; Parent et al. 2000). Moreover, in *Sander lucioperca*, a perciform fish species, it is shown that leptin induces the brain expression of *gnrh2* (Schaefer and Wuertz 2016), and in goldfish, *gnrh2* has been reported to be a downstream transcriptional target of *crh* (Kang et al. 2011). In response to feeding, *crh* and *gnrh2* have been recently reported to have similar expression patterns in *Schizothorax davidi* (another Cypriniformes species) (Yuan et al. 2021). However, other anorexigenic genes were also found showing strong response to feeding, which can be independent of leptin mediated transcriptional regulation (Yuan et al. 2020).

In this study, we set out a stepwise approach with the aim of unravelling potential regulatory connections by which leptin signal can control the expression of *cart*, *crhb* and *gnrh2* genes in zebrafish brain. We have already established this approach using qPCR analysis, co-expression databases and de novo prediction of TF binding sites to identify GRNs underlying different biological processes such as musculoskeletal morphogenesis, pigmentation and regeneration in teleost fish (Ahi and Sefc 2017, 2018; Ahi et al. 2019b).

We identified 5 genes co-expressed with all the *cart* genes making a network of co-regulated genes with reduced expression after changes in feeding condition in only wild-type group. Among these genes, *ckmt1*, *sat1a.2* and *arg2* have been shown to be expressed in the brain of zebrafish (Singh et al. 2010; Drew et al. 2012; Lien et al. 2013) and mouse (Yu et al. 2001; Yang et al. 2008; Pfefferle et al. 2011; St-Amand et al. 2012). *Ckmt1* encodes a creatine kinase required for the transfer of high energy phosphate from mitochondria to the cytosolic carrier (creatine), spermidine N1-acetyltransferase 1, *sat1a.2*, encodes an enzyme involved in the catabolic pathway of polyamine metabolism, and arginase 2 encoded by *arg2* is an enzyme catalysing the hydrolysis of arginine to ornithine and urea. However, their potential functions at downstream of leptin signalling and their effects on feeding behaviour have not been studied. A recent study in zebrafish has demonstrated that *ckmt1* is a transcriptionally responsive gene to feeding with carbohydrate enriched diet (Ma et al. 2020).

Three of the *crhb* co-expressed genes, *chd13*, *cort* and *oxt*, respectively, encode cadherin-13, cortistatin and oxytocin/isotocin neurophysin, and are expressed in vertebrate brain (Takeuchi and Ohtsuki 2001; Unger and Glasgow 2003; de Lecea 2008; Blechman et al. 2011). Both *cort* and *oxt* have overlapping hypothalamus expression in zebrafish (Unger and Glasgow 2003; de Lecea 2008; Blechman et al. 2011), but so far *chd13* expression in the hypothalamus has only been investigated in mammals (Forero et al. 2017; Kiser et al. 2019). Interestingly, all three genes have related functions in their role in vertebrates behaviour, such as effects on locomotor activity and feeding behaviour (Spier and de Lecea 2000; Onaka et al. 2012; King et al. 2017; Kiser et al. 2019). It should be noted that *crh* function is also associated with behavioural changes such as increased anxiety-like behaviour, reduced aggressive behaviour, changes in locomotor activity and reduced feeding, which result in anorexigenic action in teleost fish (Matsuda 2013). Interestingly, a study in zebrafish with loss of function in leptin gene (*lepa*) has shown increased anxiety-like behaviour with reduction in aggressive behaviour (Audira et al. 2018). These findings might indicate that leptin signal exerts its anorexigenic effects on zebrafish behaviour through hierarchical regulation of *crhb* co-expression network genes.

Among the top 5 genes co-expressed with *gnrh2*, we only found one gene, *pmchl*, to show higher expression level in wild-type than the mutant at normal feeding condition. *Pmchl* encodes a pro-melanin concentrating hormone-like protein which is expressed together with its paralogous gene (*pmch2*) in zebrafish hypothalamus (Berman et al. 2009). While the orthologous gene to *pmch2* is also present in mammals (*PMCH*) and has role in appetite regulation, *pmchl* gene only exists in fish and its potential role in feeding remained unexplored (Berman et al. 2009). However, an expression study of both *pmch* genes in the brain of the flatfish (*Platichthys stellatus*) has revealed differential regulation of *pmch1* between fasting and feeding groups indicating its potential role in appetite regulation in fish (Kang and Kim 2013).

Among the transcription factors (TFs) predicted upstream of the *cart*-coexpression module, only specificity protein3a, *sp3a*, has shown expression patterns similar to the co-expressed members of the network with reduced expression during fasting compared to normal feeding condition in wild-type zebrafish (Figs. 2d and 3a). This suggests that *sp3a* is potentially a direct upstream regulator of the *cart*-coexpression module. On the other hand, the loss of this pattern in the *lepr* mutant indicates that *sp3a* expression is under the control of leptin signal in zebrafish brain during fasting. *sp3a* encodes a transcription factor belonging to Sp1 related family genes which can have bi-functional roles in stimulating or repressing the transcription of numerous target genes (Majello et al. 1997). In humans, it has been shown that Sp3 (encoded by an mammalian orthologue of *sp3a*) and Sp1 can have synergistic or opposite regulatory effects on transcription, while binding to the same regulatory element upstream of genes playing a role in lipid metabolism and the pathogenesis of obesity in adipose tissues (Barth et al. 2003; Hoffmann et al. 2013). Interestingly, it has already been demonstrated in mammals that leptin signal can enhance the regulatory effects of Sp1 and Sp3 on the transcription of their target genes (Lin et al. 2006; García-Ruiz et al. 2012). This enhancement in transcriptional regulation can be through leptin-mediated increase in the binding affinity of Sp1 and Sp3 to their regulatory elements on the promoters of their target genes or via direct expression induction of Sp1 and Sp3 by leptin signal (Lin et al. 2006; García-Ruiz et al. 2012). In addition to our results, the findings in mammals imply a potential regulatory axis in which leptin activity is required for *sp3a* expression and subsequently *sp3a* acts as upstream transcriptional regulator of the *cart*-coexpression module. The potential role of *sp3a* during feeding and appetite regulation has not been investigated. A recent study in zebrafish has demonstrated though that the presence of sp1/sp3 binding site is essential for transcriptional regulation of *elovl5* gene (Goh et al. 2020), which encodes an enzyme involved in diet induced obesity in vertebrates (Wang et al. 2006).

We did not find expression pattern similarities between members of *crhb*-coexpression module and their predicted upstream TFs (Figs. 2e and 3b). This could indicate that the expression regulation might be mediated indirectly through other TFs or the predicted TFs might acquire different regulatory capability due to post-translational changes. For instance, it has been shown in mice that functional leptin signalling is required for Mafa (V-Maf musculoaponeurotic fibrosarcoma oncogene homolog A), to be capable of nuclear localization (without effects on *Mafa* expression though) and to exert its transcriptional regulatory effects (Harmon et al. 2009). Since the expression pattern of *mafa* observed in our study is similar between the two genotypes, it is likely that mafa nuclear localization (rather than its expression) is affected in the absence of leptin signal and thus its potential regulatory effects on *crhb*-coexpression module are lost. Another potential scenario could be transcriptional regulation of *crhb*-coexpression module again by *sp3a* (identified as potential upstream regulator of the previous module as described above) through binding to rap1 element at upstream sequences of the members of *crhb*-coexpression module. Again, the overall expression pattern of *rap1a* is not different between the genotypes, but it is already known that Sp3 and Sp1 can bind to Rap1 binding site in mammals and activate the transcription of Rap1 target genes (Simon et al. 1997).

Among the TFs predicted upstream of *gnrh2* and *pmchl*, we did not find any TF showing similar expression pattern to *gnrh2* and *pmchl* and with higher expression level in wild-type than the mutant at the normal feeding condition (Figs. 2f and 3c). However, we found binding sites for rap1 and sp1 on the regulatory sequences of both *gnrh2* and *pmchl*, which might again indicate trans-activation of these genes through *sp3a* (because of its affinity to bind to both rap1 and sp1 binding sites). Another TF that showed opposite expression pattern to *sp3a*, *gnrh2* and *pmchl* was *krox24* or *egr1* (early growth response 1), with higher expression levels in the mutant than wild-type at the normal feeding condition (Figs. 2f and 3c). In mammals, leptin and *Krox24*/*Egr1* are shown to stimulate each other’s expression as reciprocal transcriptional regulators (Bjørbæk et al. 2001; de Lartigue et al. 2010; Kim et al. 2019). Interestingly, several studies have already demonstrated that Sp1/Sp3 can compete with Krox24/Egr1 on their binding sites during the transcriptional regulation of their common target genes (Bahouth et al. 1997; Thottassery et al. 1999; Barroso and Santisteban 1999; Du et al. 2000; Tan et al. 2003; Zhang and Liu 2003; Bedadala et al. 2007). This binding competition can be favoured towards transcriptional repression or induction of a target gene following an increase in abundance of Krox24/Egr1 or Sp1/Sp3, respectively. Therefore, the reduced expression of *gnrh2* and *pmchl* in the mutant at normal feeding condition might be a result of increased expression of *krox24/egr1* which acts as transcriptional repressor. Moreover, it has been recently shown that ghrelin, an antagonist of leptin, can act as an upstream regulator of *krox24/egr1* in zebrafish brain (Blanco et al. 2020).

We summarised our results by depicting a potential leptin-dependent gene regulatory network in zebrafish brain, which might be affected by changes in feeding condition (Fig. 4b). However, it is important to note that the current study is conducted with a limited sample size and sex-dependent differences cannot be ruled out. Future studies with larger sample size including enough number of individuals from both sexes as well as overfeeding treatment are required to fully explore the expression dynamic of the potential GRN. Further functional investigations, including high-throughput methods such as transcriptome, can also provide a more comprehensive map of all potential GRNs in zebrafish brain.

## Conclusions

The study provides first evidence for the existence of a complex gene regulatory network in the brain at downstream of leptin signal which is involved in regulation of feeding in zebrafish. This network consists of transcription factors such as *sp3a* and *krox24/egr1* and their downstream genes, such as *cart* gene family, *crhb*, *cort*, *oxt*, *pmchl* and *gnrh2* (forming coexpression modules), which are involved in behavioural and metabolic control of feeding in fish. The impaired leptin signal led to reduced expression of an upstream regulator of the network, *sp3a*, which in turn caused reduced expression of the downstream network genes in brain. These regulatory effects seem no longer to be maintained after fasting in zebrafish brain.

## Supplementary Information

Below is the link to the electronic supplementary material.

Supplementary Data 1 qPCR primers and statistical results (XLSX 28 KB)

## Data Availability

All the data represented in this study are provided within the main manuscript or in the supplementary materials.

## Abbreviations

*cart*: Cocaine- and amphetamine-regulated transcripts
*crh*: Corticotropin-releasing hormone
*gnrh2*: Gonadotropin-releasing hormone 2
GRN: Gene regulatory network
*lepr*: Leptin receptor
qPCR: Quantitative real-time PCR
RQ: Relative expression quantities
TF: Transcription factor
